# For-Profit Hospitals Out of Business? Financial Sustainability During the COVID-19 Epidemic Emergency Response

**DOI:** 10.34172/ijhpm.2020.67

**Published:** 2020-05-04

**Authors:** Florien Margareth Kruse, Patrick P.T. Jeurissen

**Affiliations:** ^1^IQ Healthcare, Radboud University Medical Center, Nijmegen, The Netherlands.; ^2^Ministry of Health, Welfare and Sport, The Hague, The Netherlands.

**Keywords:** For-Profit Hospitals, COVID-19, Epidemics, Financial Resilience

## Abstract

This perspective argues that for-profit hospitals will be heavily affected by epidemic crises, including the current coronavirus disease 2019 (COVID-19) outbreak. Policy-makers should be aware that for-profit hospitals in particular are likely to face financial distress. The suspension of all non-urgent elective surgery and the relegation of market-based mechanisms that determines the allocation and compensation of care puts the financial state of these hospitals at serious risk. We identify three organisational factors that determine which hospitals might be most affected (ie, care-portfolio, size and whether it is private equity [PE]-owned). In addition, we analyse contextual factors that could explain the impact of financial distress among for-profit hospitals on the wider healthcare system.

## Background

For-profit hospitals pursue returns on equity. They do so with a business model that, firstly, relies on high and stable cashflow (for which healthcare is well-known) and, secondly, targets more lucrative sectors such as elective surgeries for less complex patients.^1^ The coronavirus disease 2019 (COVID-19) pandemic suddenly makes this business model much more risky. In this perspective we argue that the for-profit hospital sector will see considerable changes. We identify three organisational factors that determine the financial resilience of for-profit hospitals, and we discuss that certain healthcare systems are more vulnerable than others to financial distress in the for-profit hospital sector.

The COVID-19 outbreak has caused a surge of patients seeking medical care in numerous hospitals, especially in facilities with many acute care beds. Other hospitals might be confronted with the opposite fate: they are practically empty. In several countries, it is notable that particularly private, for-profit hospital are under severe pressure.^2^ For-profit hospitals tend to focus on non-acute elective care, more so than other hospital ownership types.^1,3,4^ They have therefore experienced a drop in demand. First, non-acute care had to be put on hold to free up human resources, facilities, beds and equipment materials. Second, suspending non-acute care minimises the spread of severe acute respiratory syndrome coronavirus 2 (SARS-CoV-2). Third, the supply of personal protective equipment is limited so it has to be used where it is most needed, and non-acute care therefore had to make way for acute care. Fourth, in various healthcare systems, the public emergency response to COVID-19 has side-lined market-based mechanisms (eg, patient choice and fee-for-service contracts) and for-profit hospitals relied on these mechanisms. The collapse in stock market prices for for-profit hospital chains reflects the financial hardship the sector is experiencing. For example, the share prices of Spire Healthcare Group, Community Health Systems, and Tenet Healthcare Corporation fell by approximately 60% from 20/2 and 20/3.^5^ HCA Healthcare and Universal Health Services saw their share prices almost halved.^5^ The price decrease of publicly-quoted (PQ) healthcare chains were steeper than the decline of the S&P 500, which was -28.6% between 20/2 and 20/3. And although the stock market has recovered somewhat, HCA, the bellwether of the industry, skipped share repurchases and dividend payments.^6^

## Definition

We focus specifically on for-profit hospitals. For-profit hospitals are different from public and non-profit entities insofar as that they can distribute their net earnings to their shareholders and hold all residual claimant rights. Non-profits have to comply with a non-distribution constraint and are expected to serve the interests of ‘beneficiary stakeholders.’^7^

## Financial Resilience For-Profit Hospital Sector: ThreeOrganisationalFactors

We define three organisational factors that determine the financial resilience of for-profit hospitals in the specific context of pandemics such as the outbreak of COVID-19: care portfolio, size and ownership.

### Care Portfolio

The impact of the epidemic on for-profit hospitals depends strongly on their specific care portfolios. These range from more mixed portfolios to the almost exclusive provision of outpatient treatments (ie, outpatient hospitals and independent treatment centres, known as ‘ambulatory surgical centers,’ ASCs, in the United States). Hospitals that only provide non-acute care experience a larger drop in demand. Even those for-profit hospitals that provide acute care beds tend, financially, to rely heavily on elective treatments.^1^ For-profit hospitals will lose a great chunk of their revenue during the COVID-19 response, eroding their profit margins. Even if for-profit hospitals can compensate for the financial setback by increasing their capacity of acute care beds, this may not be a lucrative business and serve only to cover costs. In some countries, acute care beds are often the most expensive service to provide.^8,9^

### Size

For-profit hospitals come in all shapes and sizes. The large, often chain-affiliated, hospitals are more resilient than small, often sole-proprietorship, hospitals because they often have more reserves and are able to cross-transfer money from different businesses. Small-scale hospitals do not have that luxury and tend to be less profitable,^10^ which makes them more vulnerable to financial default because they might have not been able to build up reserves.

### Private Equity Owned Versus Publicly-Quoted and Owner-Managed Hospitals

We argue that the private equity (PE)-owned hospitals are especially at risk of default on their payments compared to PQ and owner-managed (OM) entities because PE firms tend to take higher financial risks.^11^ The PE-owned hospitals are often the most debt-inflated providers.^12,13^ Because of their high debt-to-equity ratios, they depend on a constant cashflow. Moreover, PE firms usually have short time horizons: they seek to sell companies with a decent profit after a limited period. This risk-embracing short-term strategy may turn against PE-owned hospitals in an epidemic crisis.

OM hospitals tend, at least in theory, to be more risk-averse and to have longer time horizons for running their businesses because the investors are involved in the daily management of the company and are more ‘emotionally’ committed. For example, physicians who own their hospitals want to earn a decent financial return but are also incentivised to maintain a financially sustainable business over a long period.

PQ hospitals have to comply with stricter financial transparency and accounting regulations, which may make these hospitals’ finances more robust than others’. However, the financial status of PQ hospitals still varies (see Table 1). For example, HCA has a solvency rate of -13% in 2018 and Tenet Healthcare has a rate of -1%, whereas Universal Health Services has a rate of 48%.^5^ (This may however be explained by the previous involvement of PE in HCA and Tenet Healthcare^13^). Due to the pandemic, it will be more difficult for PQ hospitals to raise funds on the stock market. PQ hospitals with high debts and plummeting share prices are therefore confronted with a double burden. Table 1 also shows that PQ hospitals are especially active in the United States.

**Table 1:** Financial Status Pre-COVID-19 of the Main Publicly-Quoted Hospital Chains

	**Operating Revenue (Turnover) the Last Available Year ( US$Billion )**	**Average Annual Profit Margin (2010-2018) (EBT Over Operating Revenue)**	**Solvency Rate (Debt/Asset) 2018**	**Global Outreach** ^a^
HCA Healthcare, Inc.	46.7 (2018)	10.0%	-12.6%	US, UK
Ramsay Health Care Limited	8.0 (2019)	8.2%	26.3%	AU, DE, UK, ID, MY, HK, IT, FR, DA
Tenet Healthcare Corporation	18. 3 (2018)	1.3%	-0.5%	US
Community Health Systems, Inc.	14.2 (2018)	-1.6%	-9.7%	US
Universal Health Services, Inc.	10.8 (2018)	10.9%	47.8%	US, UK
Spire Healthcare Group PLC	1.2 (2018)	12.3%	31.3%	UK
Fresenius SE & CO KGAA	31.3 (2016)	10.8%	44.7% (2016)	DE, ES

Abbreviations: US, United States; UK, United Kingdom; AU, Australia; DE, Germany; ID, Indonesia; MY, Malaysia; HK, Hong Kong; IT, Italy; FR, France; DA, Denmark; ES, Spain; COVID-19, coronavirus disease 2019; EBT, earning before taxes. Source: Bureau van Dijk.^5^

## Financial Resilience to an Epidemic Shock

We argue that the care portfolio of for-profit hospitals is the most influential factor for their financial resilience, followed by size and ownership. Figure presents a schematic outline of these factors. The corners indicate the combination of organisational factors of for-profit hospitals which determine their financial resilience in an epidemic crisis. (Because small-scale PE-owned hospitals are very rare, or non-existent, we left these corners out). The corners include scores. One (1) indicates the most vulnerable organisational form and six (6) indicates the least vulnerable. Thus, small-scale OM hospitals that focus strongly on outpatient treatments are most at risk (corner 1 in Figure). The for-profit hospitals that are on the safer side of the spectrum (corner 6) are providers that are (*i* ) not owned by a PE firm, (*ii* ) that provide a mixed care-portfolio and (*iii* ) are relatively large.

**Figure:**
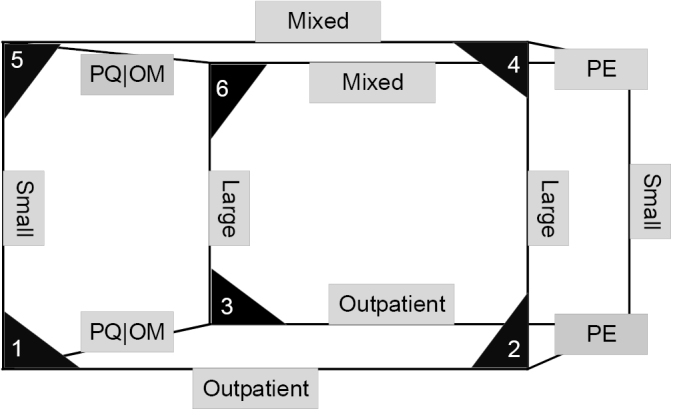
Schematic Outline Financial Resilience For-Profit Hospitals. Abbreviations: PE, private equity; OM, owner-managed; PQ, publicly-quoted.

## Context Matters

The impact on the healthcare system of for-profit hospitals getting into financial trouble is context-dependent. Table 2 outlines the factors that determine the vulnerability of different healthcare systems and we have selected a few countries to illustrate this.

**Table 2:** Contextual and Institutional Factors Related to the Financial Resilience of For-Profit Hospitals and the Vulnerability of Healthcare Systems

	**United States**	**United Kingdom**	**Spain**	**Germany**	**Australia**	**Poland**
1. COVID-19 prevalence on 29 April	+++	++	+++	++	-	+
	1 012 583 cases, 58 355 deaths^15^	161 145 cases, 21 678 deaths^15^	210 773 cases, 23 822 deaths^15^	157 641 cases, 6115 deaths^15^	6738 cases, 88 deaths^15^	12 218 cases, 596 deaths^15^
2. Private sector involvement in COVID-19 outbreak	++	++	+++	++	+	--
	For-profit hospitals are in the COVID-19 frontline. They are legally obliged to treat patients in need for emergency care, regardless of ability of pay.^16^ The increase of publicly funded patients (uninsured, Medicaid, Medicare) will decrease profit margins because rates are substantially lower than private rates.^17^	The NHS has block-bought the capacity of the private sector. The private sector receives a cost-covering price.^18^	The government has taken a more intrusive approach of placing private healthcare providers under their control, assuming charge of their buildings, human resources and material resources (eg, respirators).^19^	For-profit hospitals has joined forces against COVID-19 alongside public and non-profit hospitals.^20^ German hospitals receive a compensation of €560 per day for an empty bed. The government in addition made a federal budget available for ICU beds of €50 000 per bed.^21^	The private hospital sector helps during the epidemic crisis on a cost recovery basis after they warned the government that for-profit hospitals might need to close as a result of the suspension of most non-urgent elective surgeries.^22-24^ The private sector receives a cost-covering price.	The involvement of for-profit hospitals has been limited or blocked by the government.^25^
3. Share of for-profit hospitals in in-patient care	+	-	+	++	+	+
	17.0% share in beds (2016)^26^	3.3% share in acute beds (2014)^27^	19.1% share in beds (2017)^26^	30.4% share in beds (2017)^26^	18.9% share in beds (2016)^26^	12% share in beds (2016)^28^
4. Share of for-profit hospitals in outpatient care services	++	-	+	+++	++	+++
	The number of ASC (generally for-profit) has increased by an average annual rate of 1.0% between 2012 and 2016.^29^ 5603 ASCs were providing treatments to Medicare patients in 2017.^29^	In England, 6% of all NHS elective activity are done in ASCs (2017-2018).^30^ However, they also provide care to private patients, hence, they play a larger role in the healthcare system than the 6%.	The private sector has 29% of surgical interventions in outpatient care in 2015.^31^	Most outpatient treatment centres are run by private practices. Yet, a small proportion of these treatments are provided by hospitals.^32^	The private sector provides most of the outpatient care: in 2015, 8001 outpatient care specialists work in a private practice and 3745 in public hospitals.^33^	Specialist outpatient care is predominately provided by the private sector.^28^
5. PE-owned hospitals	++	±	±	+	+	++
	PE investments expanded significantly. In 2018, 855 deals worth $100 billion were made. Leverage buyout increased from 5% in 2000 to 14% in 2018%.^13^	Although the involvement of PE funds in the UK seems to be more active in the long-term care sector. They are also active in the hospital sector. Eg, Circle Health belongs to a PE firm. In 2019, they are merging with BMI healthcare.^34^	The largest private hospital chain (Quironsalud) was bought from two different PE firms to the German hospital chain, Fresenius.^35^	Between 2013 and 2018, 22% of the PE acquisitions in the healthcare sector were in the hospital sector. These accounted for 27 hospitals.^36^	Although it is difficult to obtain information for Australia, Australia is together with China and India the country with the highest activity of PE firms in the Asia-Pacific.^37^ In 2019, the second largest private hospital chain was taken over by Brookfield Asset Management.^38^	The expansion of the private sector in Poland caught the attention of PE firms.^14^ Eg, the largest private provider of outpatient healthcare was owned by a PE company (Mid Europa Partners).
6. Market consolidation in-patient care (if possible, private sector specific)	++	++	+++	++	++	±
	90% of Metropolitan Statistical Areas had highly concentrated hospital markets in 2016.^39^	The four largest private hospital chains cover 60% of the total independent hospital sector.^27^	The private hospital market has especially in Barcelona strongly consolidated over the years.^40^	40% of hospitals operate in highly concentrated hospital markets.^41^	The four largest private hospital groups own ± 80% of the for-profit hospitals.^42^	The total hospital market is fairly concentrated.^43^

Abbreviations: COVID-19, coronavirus disease 2019; NHS, National Health Service; PE, private equity; ASC, ambulatory surgical centre; ICU, intensive care unit. +++ very high, ++ high, + somewhat high, ± neutral, – somewhat low, - - low, - - - very low.

The impact of COVID-19 on the for-profit sector differs by country. Firstly, the infection rate of COVID-19 varies by country. Australia has fared relatively well, whereas Spain and the United States have been more severely affected. Secondly, the role that for-profit hospitals have either been assigned or taken on voluntarily during the COVID-19 outbreak also varies by country. For instance, Spanish for-profit hospitals have been under governmental control since the epidemic broke out, and the impact of this measure on the private sector is difficult to predict. By contrast, the private sector in Poland has been side-lined; if they receive neither a cost-base recovery rate nor a loss-making rate during this period, it could deal a severe financial blow to the sector. Other countries, including the United kingdom and Australia, do receive a cost-base recovery rate. The question then is whether the cost-base recovery rate will be sufficient to avoid financial difficulties in the long run. In the United States, for-profit hospitals have a different problem: during the crisis, they will be treating more patients who are covered by low-margin Medicaid reimbursement rates. US hospitals also face an especially high burden of debt, likely due to the high level of PE ownership in the United States for-profit sector.

The extent to which different healthcare systems depend on the for-profit sector for providing in-patient care differs. The for-profit sector’s share of in-patient beds ranges from 30% in Germany to 5% in the United Kingdom. In addition, in most healthcare systems there is high degree of market consolidation in in-patient hospital care, which makes healthcare systems like Germany’s relatively vulnerable when a for-profit chain with a large market share faces financial distress. Although the risk of default may be lower among large multi-hospital chains, if they fail, the impact on the wider healthcare system is much more serious. The much smaller for-profit hospital sector in the United Kingdom is at less risk and plays a limited role versus other healthcare systems, however the market consolidation in the United Kingdom for-profit sector is substantial: when one large chain defaults on its payments, it may significantly disrupt the entire for-profit sector. In other countries, such as Germany and Australia, debts associated with PE ownership are also a serious threat to for-profit in-patient hospitals.

Most countries in Table 2 lean heavily on the private sector to provide outpatient care. (In the United Kingdom its influence is much smaller, however). This may make the ASC sector relatively vulnerable to financial distress in the for-profit sector. Although it is challenging to obtain data on the ownership status of the different ASCs in each country, we do know that in the United States and Poland a number of ASC chains are PE-owned,^13,14^ and this could make these sectors more vulnerable.

## Conclusion

In various countries, the public sector has turned to the for-profit sector for help, but prices with a viable profit-margin are deemed publicly unacceptable in many countries during this crisis response. We argue and conclude that this virus will, mostly likely, weaken the position of the for-profit hospital sector, just as the Great Depression did in the 1930s.^1^ (Although 90 years ago, social healthcare insurance was far more limited in most countries). For most, their revenue decreases by the day and options to attract capital are limited – private investors are cautious and it can be challenging to acquire additional bank loans. The financial condition of the hospitals prior to the COVID-19 outbreak is an important determinant of how able they are to absorb the external financial shock. One of the lessons that for-profit hospitals and regulators can learn from this crisis is that for-profit hospitals should set aside some reserves for a rainy day because black swans also exist in hospital care. Likewise, we should be wary of hospital business models that have high debt-to-equity ratios.

The disruptive effect of COVID-19 will highlight which for-profit hospitals lack the financial resilience to outlive this crisis. In this perspective we state that (1) some for-profit hospital forms are more vulnerable than others (see Figure), and (2) that some healthcare systems are more vulnerable to a fragile for-profit hospital sector (Table 2). We therefore want to make policy-makers aware that the pandemic may lead to significant changes both within the for-profit hospital sector and in relation to the broader healthcare system.

The financial fragility of the for-profit hospital sector can set three things in motion:

Some hospitals might have to close. This requires a governmental response, either by bailing them out, nationalising the hospitals, or coordinating their default.^44^

PE firms might seize this opportunity to buy out for-profit hospitals, but the desirability of these firms infiltrating the healthcare system is questionable.^45^

For-profit hospitals that are most likely to default on their payments may be acquired by other hospitals, leading to a more consolidated hospital market. A consolidated hospital market does not lead to lower pricing and may not enhance value.^46^

Policy-makers may want to conduct an assessment, like that in Table 2, of the likely impact on the wider healthcare system of financial distress in the for-profit hospital sector.

## Acknowledgments

Our gratitude goes to Nicholas Crawford who helped us to improve the text.

## Ethical issues

Not applicable.

## Competing interests

Authors declare that they have no competing interests.

## Authors’ contributions

FMK initiated, conceptualised, and drafted the manuscript. PPTJ critically revised the manuscript for important intellectual content.

## Authors’ affiliations

^1^IQ Healthcare, Radboud University Medical Center, Nijmegen, The Netherlands. ^2^Ministry of Health, Welfare and Sport, The Hague, The Netherlands.
